# Synthesis and evaluation of corrosion inhibitory and adsorptive properties of N-(β-ethoxypropionitrile-N,N-bis(2-hydroxyethylethoxy) fatty amide

**DOI:** 10.1098/rsos.211066

**Published:** 2021-09-29

**Authors:** Gulmira Rakhymbay, Raigul Jumanova, Khaisa Avchukir, Yeldana Bakhytzhan, Akmaral Argimbayeva, Bibisara Burkitbayeva, Mirgul Turmukhanova, Florence Vacandio, Adewale Adeloye

**Affiliations:** ^1^ Center of Physical Chemical Methods of Research and Analysis, Al-Farabi Kazakh National University, 71 al-Farabi Ave., Almaty 050040, Kazakhstan; ^2^ Aix-Marseille University, CNRS, MADIREL UMR 7246, 13397 Marseille cedex 20, France

**Keywords:** ester amide derivative, steel, corrosion, inhibition efficiency, adsorption, impedance

## Abstract

The present study reports a synthetic condensation process of a vegetable oil (waste) reacted with triethanolamine, maleic anhydride and acrylonitrile in (1 : 1.2 : 2 : 1) mole ratios to obtain N-(β-ethoxypropionitrile)-N,N-bis(2-hydroxyethylethoxy) fatty amide as a major inhibitory product. Corrosion property of steel in a 3% NaCl solution in the presence of a potential inhibitor was investigated using weight loss, potentiodynamic polarization and electrochemical impedance spectroscopy (EIS) methods. These methods gave consistent results, from which it is noticeable that inhibition efficiency increases with the increasing concentration of the inhibitor. Gravimetric studies show an increase in the sample mass at an inhibitor concentration of 10 mM, indicative of adsorbed film formation on the surface. The polarization curve results showed that the compound demonstrates itself as an anodic-type inhibitor. A rise in polarization resistance values in the EIS measurements also confirmed that the compound acts as an effective inhibitor of steel corrosion. Furthermore, the R(CR)(QR) equivalent circuit was used to interpret the results obtained in the investigation of the corrosion behaviour of steel in solution with an inhibitor. The standard adsorption free energies calculated from the Langmuir isotherm indicate that adsorption takes place by physical and chemical mechanisms. The presence of adsorbed protective film was confirmed by FT-IR spectrum and SEM micrographs.

## 1. Introduction

High mechanical strength and low cost are important characteristics that ensure widespread use of metallic materials in the marine environment, particularly in production, processing, transportation and construction of underground pipelines, fuel tanks and heat exchangers [1–5]. Nevertheless, the high content of aggressive sodium chloride in marine water contributes to a decrease in the corrosion resistance, strength and workability of many metals and alloys. Among the various methods that minimize these adverse effects, steel inhibition by organic molecules is one of the most extensively used methods because of its low cost and stability [6–10]. There are many works that have been devoted to the use of effective heterocyclic and/or heteroatomic containing organic compounds that improve the anticorrosion properties of steel in sodium chloride solution. The presence of heteroatoms in particular the (O, S, N, P), aromatic rings and multiple bonds with π-electrons in these inhibitors contribute significantly to the formation of passive barriers on metal surface, thereby blocking the active sites of corrosion [1,11–16]. However, reports have also shown that not all of the proposed inhibitors are environmentally friendly and cost-effective.

Green plants for instance have been used in different numbers of recent technology advancement including solar energy generation, synthesis of nanoparticle and water purification, to mention but a few, since it is well known that plants are readily available, abundant, non-toxic, biodegradable, low cost and free from harmful emissions [17–19]. Reduction in air pollution and environmental toxins has been adduced as important advantages of natural product materials [20].

Vegetable oils have been one of the materials used in the production of biodiesel due to the presence of a significant quantity of free fatty acid, about 5 wt% [21,22]. In addition, the use of vegetable oils has also been employed by different workers to obtain important inhibitors as found in polycondensation reaction synthesis for fatty amide derivatives [23–26]. Researchers developed vegetable seed oil-based polyols, such as linseed polyol polyurethane/TEOS/fumed silica nanohybrid composites [27], polyurethane fatty amide/TEOS [28] polyetheramide resin (CPETA) [29], which showed good physicomechanical, anticorrosive properties in different corrosive media.

**Figure 1.**
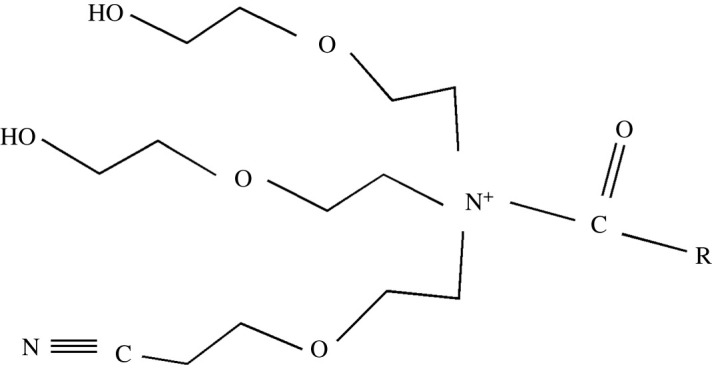
N-(β-ethoxypropionitrile)-N,N-bis(2-hydroxyethylethoxy) fatty amide.

Nowadays, theoretical studies are very important in corrosion studies. In this work, inhibitor for steel corrosion, a waste vegetable oil as a source of free fatty acid and functionalized with tris-(2-hydroxyethyl) amine, maleic anhydride and acrylonitrile as components were investigated (figure 1). The choice of a representative of compounds of this class as a potential candidate for a highly effective steel corrosion inhibitor is reasonable due to its facile synthetic method, low cost and presence of oxygen and/or nitrogen heteroatoms containing either double or triple bonds in the chemical structure. The inhibition efficiency of the compound was primarily investigated by weight loss and further confirmed by electrochemical methods. Other studies such as the surface morphology and adsorption behaviour of the inhibitory compound were determined using scanning electron microscopy (SEM).

## 2. Experimental procedure

### 2.1. Chemicals

Waste vegetable oil was obtained from Al-Farabi Kazakh National University, Almaty, Kazakhstan. Methanol, acetone, tris-(2-hydroxyethyl) amine and maleic anhydride were purchased from Merck, Germany and NaOH was purchased from Sigma Aldrich UK. All chemicals are used as supplied without any other purification. Protection efficiency of inhibitor was determined at different concentrations range 0.1 to 10 mM by dissolving in a 3.0 wt% NaCl. The electrolytes were prepared using analytical grade reagents (Sigma Aldrich). Gravimetric and electrochemical experiments were carried out using grade St3 steel samples containing elemental compositions (in wt%): C, 0.14–0.22; Si, 0.15–0.3; Mn, 0.4–0.65; Ni, less than 0.3; S, less than 0.05; P, less than 0.04; Cr, less than 0.3; Ni, less than 0.008; Cu, less than 0.3; As, less than 0.08; and Fe, approximately 97. In order to have a mirror surface of the samples, prior to each experiment, the samples were treated with 1200, 2000 and 2500 grade emery paper (Dexter), then degreased in ethanol and finally rinsed in distilled water.

### 2.2. Preparation of methyl ester from waste vegetable oil

**Scheme 1.**
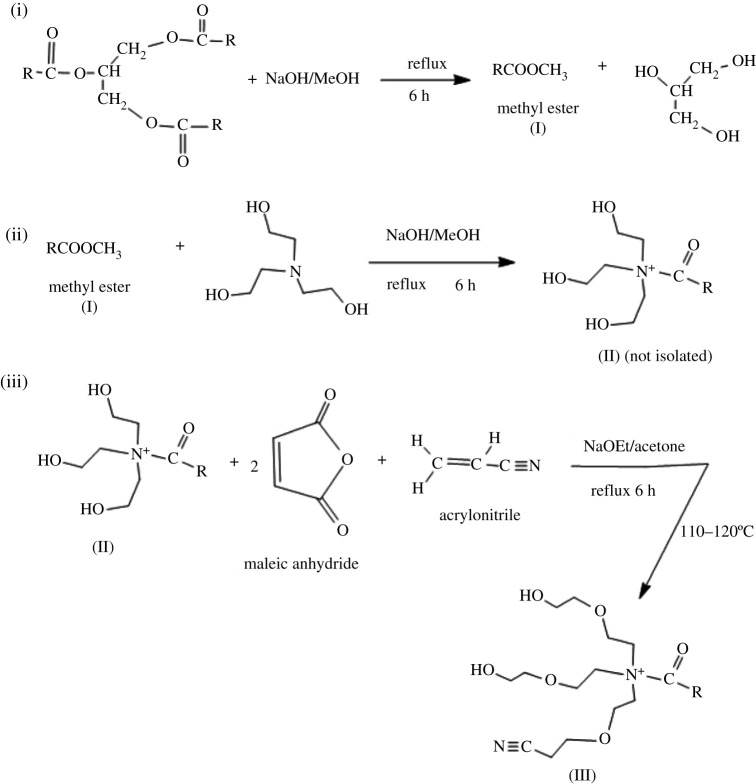
Reaction procedures for the synthesis of N-(β-ethoxypropionitrile)-N,N-bis(2-hydroxyethylethoxy) fatty amide.

The reaction followed a slight modification to the method reported by Pramanik *et al*. [30]. A 20 ml solution of 50% aqueous-methanolic sodium hydroxide (0.025 M) was added to 0.50 g vegetable oil (waste) and refluxed for 1 hour. The reaction was monitored by thin-layer chromatography. The product after reflux was extracted with chloroform (b.p. 61.2°C) and concentrated to obtain product (**I**) at 65% yield (scheme 1, (i)).

### 2.3. Synthesis of N-(β-ethoxypropionitrile)-N,N-bis(2-hydroxyethylethoxy) fatty amide

The reaction methodology followed that as reported by Pramanik *et al*. [30] with slight modifications. The reaction followed a two-step procedure as shown in scheme 1, (ii) and (iii). Product (**I**) was reacted tris-(2-hydroxyethyl) amine in (1 : 1.2) molar ratio in 20 ml solution of 50% aqueous-methanolic sodium hydroxide (0.025 M) as solvent.

The product (**II**) obtained was not isolated while aqueous-methanolic solvent evaporated. Maleic anhydride and acrylonitrile in (2 : 1) mole ratio were added, and the reaction was carried out in 20 ml sodium ethoxide and acetone (3 : 1, v/v) for good dissolution of reacting agents. The product (**III**) was obtained as a white solid. Percentage yield = 55%, TLC *R*_f_-value (0.90, in methanol), melting point at 170–180°C.

### 2.4. Evaluation of inhibition efficiency by gravimetric method

Gravimetric determination of the corrosion rate was carried out according to a unified method. Cleaned and accurately weighed steel samples in triplicates were dipped in 3.0% NaCl solution with different concentrations of the inhibitors during 120 h at T = 25°C. Scales formation are thereafter removed mechanically using a bristle brush, washed well in distilled water and ethanol, and finally dried and weighed using analytical balance OHAUS Pioneer. Weight loss tests were triply carried out for each concentration and maximum observed standard derivations obtained were less than 2%. The mean corrosion rate (*ν*) was employed to calculate inhibition efficiencies (*η*) of the inhibitor. The corrosion rates were calculated using equation (2.1) and expressed in weight loss per unit of time (g m^−2^ h^−1^) [9]

2.1 $$\nu_{m}=\frac{\Delta m}{St},$$

where Δ*m* = weight loss (g); *S* = area of the mild steel sample (m^2^); *t* = exposure time (h).

For uniform metal corrosion and based on this weighting method, deep indicator of corrosion can be obtained. The deep index of corrosion is related to the volume of collapsed metal and characterizes the penetration deep of corrosion damage over a certain time (mm yr^−1^)

2.2 $$\nu_{d}=8.760\;\cdot \frac{\nu_{m}}{\rho },$$

where *v_d_* = deep index of corrosion (mm yr^−1^); *ρ* = density of steel (g cm^−3^); 8.760 = coefficient that takes into account the measurement's translation of units.

Inhibition efficiency (*η*) evaluated from equation

2.3 $$\eta =\;\frac{\nu_{0}-\;\nu_{\mathrm{inh}}}{\nu_{0}}\times 100\;,$$

where *v*_0_ and *v*_inh_ are the corrosion rates in the absence or in the presence of inhibitor for certain concentrations, respectively.

### 2.5. Evaluation of inhibition efficiency by electrochemical techniques

Electrochemical methods (potentiodynamic polarization (PDP) and electrochemical impedance measurements (EIS)) were done on an AUTOLAB potentiostat/galvanostat PGSTAT 302 N in-built impedance analyser FRA controlled by Nova 1.11 software. The electrochemical tests were carried out in a three-compartment glass cell consisting of the steel (St3) sample as working electrode (WE), platinum counter electrode (CE) and Ag/AgCl with 3 M KCl as the reference electrode.

PDP curves were made a potential range of –300 mV to +300 mV (versus OCP) with a scan rate of 1 mV s^−1^. OCP equilibrium was reached at exposure time equal to 120 s. The various electrochemical parameters such as corrosion potential (*E*_corr_), corrosion current densities (*i*_corr_) and Tafel slopes (*β*_a_ and *β*_c_) were found by a simple extrapolation of the linear Tafel segments of the polarization curves. Inhibition efficiency, *η*, was evaluated from polarization measurements using the equation [7]

2.4 $$\eta =\;\frac{i_{\mathrm{corr}}-\;i_{\mathrm{corr}\;(\mathrm{inh})}}{i_{\mathrm{corr}}}\times 100\;,$$

where *i*_corr_ and *i*_corr(inh)_ are current densities obtained from PDP measurement in the absence or presence of investigated inhibitors, respectively.

Electrochemical impedance spectroscopy (EIS) studies were performed at a frequency range of 10^5^ to 0.1 Hz with a superimposed sine wave of amplitude 0.01 V. From the plot of Z′ versus Z″, the charge transfer resistance (*R*_ct_) and double-layer capacitance (*C*_dl_) were calculated. The inhibition efficiency was calculated using the formula [7]

2.5 $$\eta =\;\frac{R_{\mathrm{corr}\;(\mathrm{inh})}-R_{\mathrm{corr}}\;}{R_{\mathrm{corr}\;(\mathrm{inh})}}\times 100\;,$$

where *R*_corr_ and *R*_corr (inh)_ are the charge transfer resistance obtained from impedance measurement in the absence or presence of tested inhibitors, respectively. In order to determine the reproducibility of the results, all of the above studies were carried out at least three times.

### 2.6. FT-IR characterization and scanning electron microscopy

The FT-IR spectra of pure inhibitor and the inhibitor adsorbed on the steel surface were recorded using Perkin Elmer spectrometer in the frequency range from 4000 to 500 cm^–1^. The SEM analysis gives information on surface roughness/heterogeneity and determines the magnitude of corrosion damage. Presence of a chemisorption protective film proves by comparing the samples after 120 h of immersion in an electrolyte with and without inhibitor.

## 3. Results and discussion

### 3.1. Weight loss method

Gravimetric research is the most commonly used traditional method, which relies on the determination of mass loss due to corrosion from a unit area of samples of studied metals per unit time. The results obtained from the study of corrosion of samples in 3.0% NaCl in the absence or presence of inhibitor at various concentrations is as shown in table 1.

**Table 1.** Parameters of corrosion for St3 in 3.0% NaCl solution containing different concentrations of inhibitor at temperature 25°C were obtained from gravimetric tests.

inhibitor concentration (mM)	*v_m_* (g m^−2^ h^−1^)	*v_d_* (mm yr^−1^)	*η*
blank	0.110	0.121	—
0.1	0.059	0.065	46.36
0.5	0.054	0.060	50.99
1.0	0.036	0.040	67.27
5.0	0.015	0.016	86.36
10.0	mass increase		mass increase

From the measurements, it is observed that without inhibitor, the corrosion rate of St3 sample is 0.110 g m^−2^ h^−1^. The addition of a 0.1 to 5.0 mM of inhibitor in the solution led to a significant decrease in corrosion rate from 0.059 to 0.015 g m^−2^ h^−1^ respectively. So, the inhibition effect increased from 1.86 to 7.33, and the degree of protection from 46 to 86. The increase in the protective effect is most likely explained by the formation of a chemisorption film as a result of the interaction of inhibitor molecules with surface iron atoms. In the case of an increase in the concentration of the inhibitor to 10.0 mM, an increase in the mass of steel samples is observed, which confirms the formation of a more stable film with high protective functions.

### 3.2. Electrochemical methods

#### 3.2.1. Potentiodynamic polarization technique

**Figure 2.**
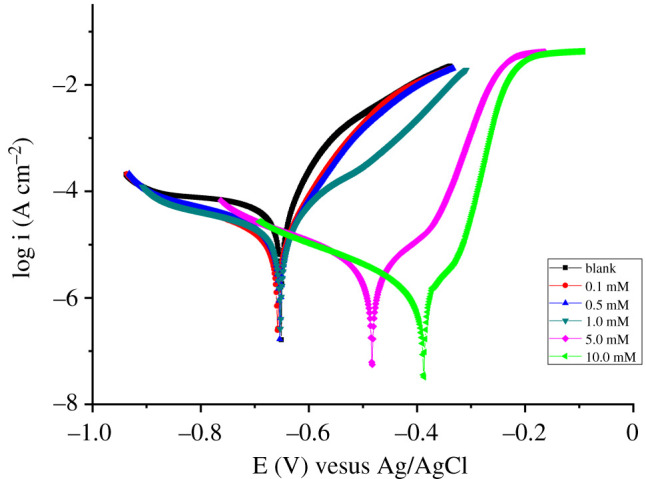
PDP curves for steel in the absence and presence of different concentrations of inhibitor in 3.0% NaCl.

The PDP method allows the study of the kinetics of anode and cathode reactions and evaluating the efficiency of the inhibitor. Polarization experiments were performed in an unmixed 3% NaCl solution in the absence or presence of various inhibitor concentrations, and the obtained polarization curves are as shown in figure 2.

The results presented in figure 2 show that the addition of inhibitor in 3.0% NaCl corrosive medium shifts the corrosion potential (*E*_corr_) toward more electropositive values and significantly reduces current density, especially the inhibitive effect is pronounced at high concentrations of the inhibitor. In comparison with the blank solution, a shift of 250 mV was observed in the *E*_corr_ of the inhibited systems. This result confirms N-(β-ethoxypropionitrile)-N,N-bis(2-hydroxyethylethoxy) fatty amide to exhibit an anodic-type inhibitory property and inhibit the anodic dissolution of iron. The useful corrosion kinetic parameters which were obtained from the Tafel extrapolation of the polarization curves are presented in table 2.

**Table 2.** Tafel polarization parameters of steel in 3.0% NaCl solution with various concentrations of inhibitor.

*C* (mM)	*b*_a_ (mV dec^−1^)	*b*_c_ (mV dec^−1^)	*E*_corr_ (mV)	*j*_corr_ 10^−6^ (A cm²)	corrosion rate (mm yr^−1^)	*η*
blank	22.73	−41.11	−650.73	7.95	0.092	
0.1	36.25	−22.41	−657.45	2.39	0.028	69.93
0.5	24.58	−16.31	−652.03	1.91	0.022	75.97
1.0	18.79	−18.96	−652.35	1.82	0.021	77.10
5.0	28.55	−29.79	−483.14	0.50	0.006	93.71
10.0	44.26	−21.04	−387.62	0.27	0.003	96.60

The data given in table 2 show a slight change of both the anodic and cathodic Tafel slopes with varying inhibitor concentrations, which indicates that the addition of the inhibitor does not significantly affect the corrosion mechanism of steel. Furthermore, the presence of the inhibitor studied caused a decrease in the current density of corrosion, thereby increasing the efficiency of inhibition where a value of 96.6 at a concentration of 10 mM was obtained. This observation suggests that the N-(β-ethoxypropionitrile)-N,N-bis(2-hydroxyethylethoxy) fatty amide effectively adsorbs on the metal surface and blocks the active centres responsible for corrosion. Thus, a higher concentration promotes the formation of a complete adsorbed film on the steel surface.

#### 3.2.2. Electrochemical impedance spectroscopic technique

**Figure 3.**
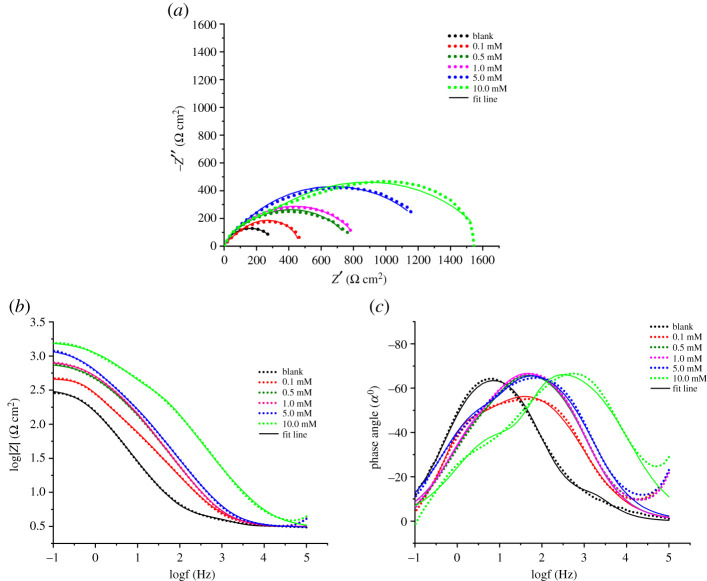
Nyquist (*a*) and Bode (*b,c*) plots for steel in 3.0% NaCl solution in the absence and presence of inhibitor at various concentrations.

EIS is one of the most significant and widely used electrochemical techniques to obtain information relating to the mechanism and kinetics of metal corrosion behaviour, as well as adsorption of inhibitors without changing the state of the metal/solution interface. The Nyquist plots and the corresponding Bode diagrams for the steel corrosion in 3.0% NaCl in the absence or presence of N-(β-ethoxypropionitrile)-N,N-bis(2-hydroxyethylethoxy) fatty amide as an inhibitor at different concentrations are presented in figure 3.

All impedance diagrams represent loops with the centre below the real impedance line, which may be related to the effect of frequency dispersion caused by adsorption of corrosion inhibitor in homogeneity and roughness of the corrosive surface [6,8]. Furthermore, the impedance spectra recorded in the presence of the inhibitor show double semicircles that correspond to two peaks in the Bode plots (figure 3*c*) the manifestations of which are noticeable at higher concentrations. Increasing the concentration of N-(β-ethoxypropionitrile)-N,N-bis(2-hydroxyethylethoxy) fatty amide increases the diameter of the semicircle and the width of the phase angle peaks, which is an indicator of the inhibition of steel corrosion by these additives due to the formation of a protective film at the metal/electrolyte interfaces.

**Figure 4.**
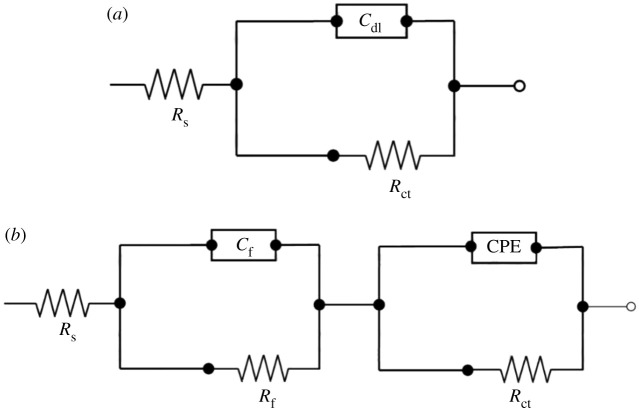
Electrochemical equivalent circuits are used for the fitting and simulation of impedance data from the blank solution (*a*) and the inhibiting solution (*b*).

Increase in the total impedance at low frequency as indicated in the Bode's plot (figure 4*b*) confirms higher protection as inhibitor concentration increased. Moreover, negative phase angle values indicate superior inhibitory properties.

In order to have accurate EIS results in the further analysis and exploration of the mechanisms of corrosion processes occurring on the surface, a suitable model of the equivalent circuit shown in the figure 4 is selected. The R(CR) equivalent circuit for bare substrate, displayed in figure 4*a*, exhibited one time constant, which is one charge transfer reaction. The adsorbed coating on the substrate was manifested as an extra time constant in the equivalent circuit (figure 4*b*), consists of a coating resistance (*R*_f_) connected in parallel with the film capacitance (*C*_f_) and a charge transfer resistance (*R*_ct_) also connected in parallel with a constant phase element (CPE); both parts of the circuit were connected in series with solution resistance (*R*_s_). The selected equivalent circuit describes the uniform distribution of the reaction sites at the interface metal/solution caused by the homogeneous diffusion of the electrolyte into the coatings [31,32]. Accurate fit of experimental data and effective representation of corrosion of heterogeneous solid surfaces were obtained by the introduction of a constant phase element (CPE). CPE has an impedance (Z_CPE_) which can be determined by the following equation [7]:

3.1 $$Z_{\mathrm{CPE}}=\frac{1}{Y_{o}{\left(\;jw\right)}^{n}},$$

where *Y_o_* is the quantity of the CPE, *j* is an imaginary unit (*j* = −1)^1/2^, *w* represents the angular frequency, and *n* describes the phase shift.

Using equation (3.2), the *Y*_*o*_ values were converted to *C*_dl_, which provided a comparison of the capacitive behaviour of different corrosion systems [33]

3.2 $$C_{\mathrm{dl}}={\left(Y_{o}R_{ct}^{1-n}\right)}^{1/n}.$$

The main impedance parameters are as shown in table 3.

**Table 3.** Electrochemical parameters and inhibition efficiency were obtained from the impedance spectra of steel in 3.0% NaCl containing various inhibitor concentrations at 298 K.

*C*_inh_ (mM)	*R*_s_ (Ω cm^2^)	*R*_ct_ (Ω cm^2^)	CPE_dl_ (*Yo*) µS s^n^ cm^−2^	*N*	*C*_dl_ (µF cm^−2^)	*C*_f_ (µF cm^−2^)	*R*_f_ (Ω cm^2^)	*R*_p_ (Ω cm^2^)	*η*
blank	3.59	251.20			631.37			251.20	
0.1	3.07	146.95	686.31	0.717	277.51	757.96	317.27	464.22	45.89
0.5	3.11	743.55	361.46	0.774	246.29	410.64	46.83	790.38	68.22
1.0	3.13	796.30	335.99	0.785	234.15	378.87	46.71	843.01	70.20
5.0	3.05	1268.56	316.88	0.759	237.26	292.30	45.83	1314.39	80.88
10.0	2.95	1507.20	148.09	0.695	76.68	23.43	139.75	1646.95	84.75

As shown in table 3, the increase of the polarization resistance *R*_p_ values and the decrease of the capacitance values of the double-layer *C*_dl_ with the increase of the inhibitor concentration are explained by the absorption of inhibiting molecules on the metal surface while replacing the water molecules present in the electrolyte. The deviation of the *N*_dl_ value from the ideal capacitive behaviour of the CPE indicates non-uniformity and surface roughness [2,34,35]. The increase of the film resistance *R*_f_ indicates the thickening of the adsorbed organic layer on the surface, which in turn allows the reduction to the number of active sites, thereby increasing the inhibition efficiency (*ɳ* %).

### 3.3. Adsorption isotherm and adsorption parameters

The study involving the influence of the chemical composition of the solution and the nature of the corrosive metal on the adsorption bond strength yield information about the mechanism of interaction of the inhibitor with the steel surface, which in turn adsorption isotherms provide excellently [7,36]. The adsorption of N-(β-ethoxypropionitrile)-N,N-bis(2-hydroxyethylethoxy) fatty amide on the surface of metal in NaCl solution was established by the most suitable Langmuir isotherm, which indicates the chemisorptive nature of the bond between the inhibitor molecules and the steel [4,6,7], the equilibrium distribution of ions of the adsorbing substance between the solid and liquid phases, and a monolayer character. The adsorbed molecules are held on the metal surface for a long time. Chemisorbed inhibitors have an after-effect and high efficiency in protective action.

The Langmuir isotherm is widely used in the determination of inhibitor adsorption process characteristics and it is expressed by the following equation [4]:

3.3 $$\frac{C_{\mathrm{inh}}}{\theta }=\frac{1}{K_{\mathrm{ads}}}\;+C_{\mathrm{inh}},$$

where *θ* is the fractional coverage of the metal surface; *C*_inh_ is the inhibitor concentration and *K*_ads_ is the equilibrium constant of the adsorption/desorption processes.

The coverage degree of the steel surface with an inhibitor (*θ*) was estimated by the equation (3.4) [36,37] in the method of EIS

3.4 $$\Theta =\frac{C_{dl(\Theta =0)}-C_{dl,\Theta }}{C_{dl(\Theta =0)}-C_{dl(\Theta =1)}},$$

where *C*_dl(*ϴ*=0)_ and *C*_dl(*ϴ*=1)_ are the double-layer capacitances (per unit area) of the inhibitor-free and entirely inhibitor covered surfaces, respectively, *C*_dl,*ϴ*_ is the composite total double-layer capacitance for any intermediate coverage *ϴ*. The value $C_{\mathrm{dl}(\Theta =1)}$ can be determined from the dependence *C*_dl_ = *f* (l/*C*_inh_) using extrapolation to the intersection with *C*_dl_ axis.

**Figure 5.**
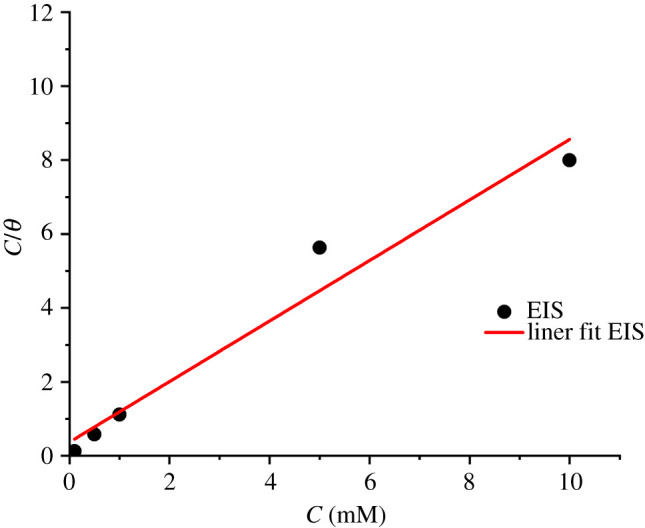
Langmuir adsorption isotherm in terms of EIS results for steel in 3.0% NaCl solution.

The Langmuir isotherm can be presented as plot of *C*/*θ* versus C as shown in figure 5.

Using the Langmuir isotherm, the equilibrium constant of adsorption/desorption processes (*K*_ads_) can be determined and be related to the standard free energy of adsorption ${\Delta G}_{\mathrm{ads}}^{\circ }$ as follows [9]:

3.5 $${\Delta G}_{\mathrm{ads}}^{\circ }=\;-\mathrm{RTln}(55,5K_{\mathrm{ads}}),$$

where *R* = is the gas constant (8314 J K^−1^ mol^−1^), T is the absolute temperature (*K*) and the value 55.55 is the concentration of water in solution expressed in mol l^−1^. The ${\Delta G}_{\mathrm{ads}}^{\circ }$ and *K*_ads_ values calculated using the intersections of straight lines on the *C*_inh_/*θ* axis are presented in table 4.

**Table 4.** Adsorption parameters of the studied inhibitor on the steel obtained from Langmuir adsorption isotherm.

method	slope	*R*^2^	*K*_ads_ (l mol^−1^)	${\Delta G}_{\mathrm{ads}}^{\circ }$ (kJ mol^−1^)
EIS	0.8187	0.9629	2694.98	−29.52

From table 4, it can be seen that the slopes of the adsorption isotherm plots deviate from unity, this may be attributed to the interactions between the organic molecules and the steel surface. High values in the correlation coefficient at 0.9629 confirmed agreement between measured data and the calculated results, as well as the correctness of the selected isotherm for the adsorption process. The adsorptive capacity of inhibitor molecules on steel is confirmed by the *K*_ads_ values. The values of the free energy of adsorption ${\Delta G}_{\mathrm{ads}}^{\circ }$ lie between −20 and −40 kJ mol^−1^ [4,9,10]. In view of this, it is assumed that the adsorption of the inhibitor on the steel surface may be physical and chemical character. As shown in table 4, all the calculated ${\Delta G}_{\mathrm{ads}}^{\circ }$ values are very close, which indicates the agreement of the measured data by all methods and the reliability of the calculations.

### 3.4. Fourier transform infrared spectroscopic studies

The inhibition of the studied substance through the formation of a protective film on the electrode was confirmed by FT-IR spectra for a pure compound inhibitor (**III**) (in powder) and its adsorbed form (sample) on the steel surface (see electronic supplementary material, data). In the former spectrum, a strong prominent vibrational frequency peak at 3302 cm^−1^ confirms the presence of O-H_str_ band of aliphatic alcohol. This region extends towards 3156 cm^−1^, a possible indication of an intermolecular hydrogen bond. Aliphatic methylene (CH_2_) group was found as two highly structured vibrational frequency bands, one at 2938–2903 cm^−1^ and the other at 2848–2725 cm^−1^. We adduced these to signify the CH_2_ groups each bonded to terminal O-H and C≡N groups respectively. The C≡N frequency vibration is known to appear very weak and broad at the specific range of 2260–2222 cm^−1^. The area in the spectral was found as multiplet band peaks. The prominent vibrational frequency band at 1737 cm^−1^ was unequivocally assigned to (C=O)_str_ possibly from the methyl ester moiety bonded to the quaternary amide group.

The amide stretch characteristic absorption is noted to occur at 1630–1690 cm^−1^. The band is conspicuously found very weak around 1635 cm^−1^, which is possibly adduced to the saturated quaternary amide bond in the molecule [30]. A diagnostic feature of the amide functional group combines both N–H and C=O band characteristics of amines and ketones, such that for primary and secondary amides, double and single spikes are present respectively and a vibrational frequency band around 1710 cm^−1^ for C=O_str_ [38]. The strong bands in the range 1487–1458 cm^−1^ are assigned to the C-H bending alkane for the methylene group. The two prominent vibration bands at 1399 and 1302 cm^−1^ were assigned to O-H bending of alcohol. The medium vibration frequency bands at 1257–1231 cm^−1^ were characteristic bands for the presence of a C-N_str_ for amine, while the strong bands at 1196–1078 and 1029–1003 cm^−1^ were assigned to C-O_str_ for aliphatic ether and primary alcohol respectively.

Comparison of the adsorbed sample FT-IR spectra with that of compound (**III**) the following important characteristics changes at the functional group and fingerprint region of vibrational frequencies were observations. At the high-frequency region, a very prominent strong intensive peak at *υ*_str_ 3302 cm^−1^ in the inhibitor (compound **III**) adduced to the presence of hydroxyl group has conspicuously disappeared resulting in a very weak broad peak at *υ*_str_ 3368 cm^−1^ in the steel-inhibitor complex. This loss of peak is indicative to the loss of (O–H) group in the molecules of the inhibitor possibly due to electron donation from the lone pair oxygenated inhibitor to form metal complexation with the steel. Another prominent feature in the FT-IR spectrum of the adsorbed protective layer shows the loss of strong multiplet bonding associated with the nitrile group (C≡N) . It was proposed that the inhibitor also may have that capacity to donate the loan pair electrons on the nitrogen to the empty *d*-orbital of the metal to form a coordination complex, though this may depend on coordination characteristics of metal present in the steel. Perhaps, cyano group frequency band observed as a weak absorption band in the region of 2200–2322 cm^−1^ may support our thinking. The presence of an ester carbonyl group previously observed at a vibrational frequency of 1742 cm^−1^ in the inhibitor (compound **III**) now appeared as a strong band at 1735 cm^−1^ in the steel-inhibitor products.

From the spectra, it was observed that the (C=O) vibrational frequency appeared intact even after adsorption of compound (**III**) on steel surface being indicative of no major participatory role in molecular bonding of the inhibitor. A band at 1097 cm^−1^ is assigned to the stretching vibrations of primary alcohol while series of peaks in the region at 1241–1162 cm^−1^ were assigned to etheryl group vibrational frequency. All the observed functional group frequency band changes in the spectrum strongly show that compound (**III**) as inhibitor successfully bound to the surface of the steel through N or O heteroatom.

### 3.5. Scanning electron microscope characterization

**Figure 6.**
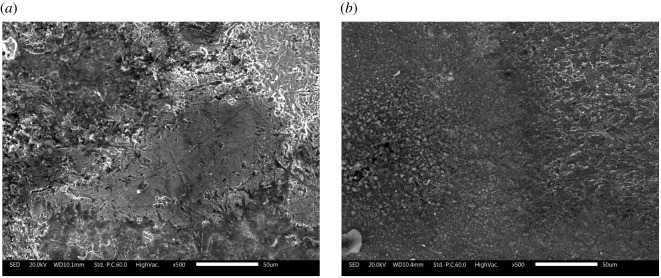
SEM micrographs of steel (*a*) after immersion in 3.0% NaCl, (*b*) after immersion in 3.0% NaCl solution with an inhibitor (10 mM).

The morphological characteristics of the obtained coatings on steel were determined by the SEM method. Figure 6 represents the micrographic images of corroded and inhibited samples with inhibitor at a concentration of 10 mM. A comparative analysis of the data showed significant differences between them: steel in an aggressive medium without an inhibitor exposed to destruction, whereas with an inhibitor, the surface was smoother and did not have any defects. Apparently, this is due to the formation of a protective film on the steel surface, which reduces the corrosion rate due to the blocking of active sites by adsorbed inhibitor molecules.

### 3.6. Chemistry of adsorption

**Figure 7.**
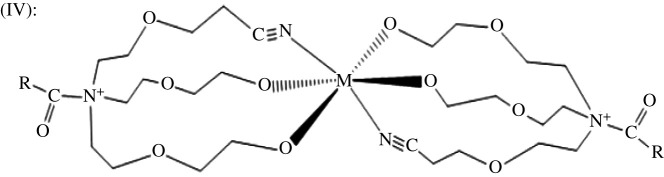
A representative structure of metal coordinated N-(β-ethoxypropionitrile)-N,N-bis(2-hydroxyethylethoxy) fatty amide.

The original structure of the inhibitor having three major reactive sites containing lone pair of electrons as shown in figure 1 depicts that it can conveniently form a complex product with steel. Generally, due to the complex chemical formulae for steel, all metal ions present having a coordination number of (+2), for instance, may possibly have oxidation reaction with two hydroxyl groups and the nitrile group of the inhibitor. Taking inhibitor structure in figure 1 to represent X in the reaction equation below: the steel surface (with metalM^2+^) reacts with X to give product structure IV (figure 7)

$$\mathrm{Steel}\;\mathrm{surface}\;(\mathrm{with}\;M^{2+}\mathrm{ions})+2\;X\to (\mathrm{IV})+4\;H^{+}.$$

## 4. Conclusion

The present work has reported the facile synthesis of N-(β-ethoxypropionitrile)-N,N-bis(2-hydroxyethylethoxy) fatty amide (compound (**III**)) obtained from waste vegetable oil and subsequently evaluated for its inhibition property for steel corrosion in 3% NaCl using chemical and electrochemical methods. From the results, the following deductions were made.

1. The gravimetric analysis shows an increase in *η* with the increased concentration of inhibitor. At a concentration of 10 mM, an increase in the sample mass was observed, indicating the formation of a protective film.
2. The data obtained from PDP pointed to N-(β-ethoxypropionitrile)-N,N-bis(2-hydroxyethylethoxy) fatty amide to exhibit itself as an anodic corrosion inhibitor. This is corroborated by the maximum shift of *Е*_corr_ to the positive region to about 250 mV. With increased inhibitor concentration, there is a corresponding increase in the inhibition efficiency up to 96.60.
3. The EIS results were in perfect agreement with the above data; the maximum value of *η* = 84.75 was achieved at the highest inhibitor concentration. The corrosive behaviour of steel in an aggressive environment was interpreted with the use of equivalent circuits described by one and two time constants for pure steel and a substrate with a protective film, respectively.
4. The adsorption of the tested inhibitor on steel was found to obey the adsorption isotherm of Langmuir. Based on the results obtained by the methods of gravimetry, PDP and EIS, the Gibbs standard free energies of adsorption was calculated, which amounted to −30.07, −31.52 and −29.52 kJ mol^−1^, respectively. The ${\Delta G}_{\mathrm{ads}}^{\circ }$ values indicate that the adsorption of N-(β-ethoxypropionitrile)-N,N-bis(2-hydroxyethylethoxy) fatty amide on the metal surface occurs by means of electrostatic interaction and chemisorption.
5. The formation of a protective film on the steel surface was confirmed by FT-IR spectral and SEM image analysis.

## Data accessibility

This article has no additional data.
