# Supplementary material for: Synthesis and evaluation of corrosion inhibitory and adsorptive properties of N-(β-ethoxypropionitrile-N,N-bis(2-hydroxyethylethoxy) fatty amide
Source: R Soc Open Sci. 2021 Sep 29;8(9):211066. doi: 10.1098/rsos.211066 (PMC8479329; doi:10.1098/rsos.211066)
Supplement: Supplementary file 1 [file rsos211066_review_history.pdf]

THE ROYAL SOCIETY
PUBLISHING

ROYAL SOCIETY
OPEN SCIENCE

# Synthesis and evaluation of corrosion inhibitory and adsorptive properties of N-(β-ethoxypropionitrile-N, N-bis(2-hydroxyethylethoxy) fatty amide

Gulmira Rakhymbay, Raigul Jumanova, Khaisa Avchukir, Yeldana Bakhytzhan, Akmaral Argimbayeva, Bibisara Burkitbayeva, Mirgul Turmukhanova, Florence Vacandio and Adewale Adeloye

**Article citation details**
*R. Soc. open sci.* **8**: 211066.
http://dx.doi.org/10.1098/rsos.211066

**Review timeline**

| | |
|---|---|
| Original submission: | 19 November 2020 |
| Revised submission: | 11 July 2021 |
| Final acceptance: | 25 August 2021 |

Note: Reports are unedited and appear as submitted by the referee. The review history appears in chronological order.

# Review History

## RSOS-202027.R0 (Original submission)

### Review form: Reviewer 1 (Ismat Ali)

**Is the manuscript scientifically sound in its present form?**
Yes

**Are the interpretations and conclusions justified by the results?**
Yes

**Is the language acceptable?**
Yes

**Do you have any ethical concerns with this paper?**
No

Reports © 2021 The Reviewers; Decision Letters © 2021 The Reviewers and Editors; Responses © 2021 The Reviewers, Editors and Authors. Published by the Royal Society under the terms of the Creative Commons Attribution License http://creativecommons.org/licenses/by/4.0/, which permits unrestricted use, provided the original author and source are credited

**Have you any concerns about statistical analyses in this paper?**
No

**Recommendation?**
Major revision is needed (please make suggestions in comments)

**Comments to the Author(s)**
This article is interested in Synthesis and evaluation of corrosion inhibitory and adsorptive properties of N-( β ethoxypropionitrile-N,N-bis(2-hydroxyethylethoxy) fatty amide. There are a lot of previous studies that investigate the potential of inhibitor materials to suppress corrosion. However, the fact that the current material has not reported previously, increased the originality of this article. The techniques used in this study are classical and commonly used for the determination of the corrosion rate.
 Nowadays, theoretical studies are very important in corrosion studies but unfortunately, it is not reported in this study.
 Despite the aim of the work is to study the efficacy of the inhibitor on corrosion behavior of st3 at room temperature, but several experiments at different temperatures should be performed to determine the thermodynamic parameters.
 The introduction section is well-written but more literature should be reported in order to make a strong case for this work.
 How authors confirmed the structure of their inhibitor. Some characterization techniques should be used.
In addition, the following points should be considered:
1) A comparison between your inhibitor and some previously reported inhibitors should be included.
2) After how long the OCP equilibrium was reached?
3) The abbreviation PVD (page 6 – line 48) should be defined.
4) Space in (page 10 line 21) fora long should be corrected.

# Review form: Reviewer 2

**Is the manuscript scientifically sound in its present form?**
No

**Are the interpretations and conclusions justified by the results?**
Yes

**Is the language acceptable?**
Yes

**Do you have any ethical concerns with this paper?**
No

**Have you any concerns about statistical analyses in this paper?**
No

**Recommendation?**
Reject

**Comments to the Author(s)**
Review for RSOS-202027

Synthesis and evaluation of corrosion inhibitory and adsorptive properties of N-$\beta$ ethoxypropionitrile-N,N-bis(2-hydroxyethylethoxy) fatty amide

This paper reports on the corrosion testing of a vegetable oil based inhibitor on mild steel. The results presents are fairly rudimentary, and a fair bit of detail is lacking. The corrosion results mainly cover weight loss, potentiodynaic polaristaion and EIS. The surface microscopy via SEM provided no suable results other than there was corrosion on the uninhibited surface compared with the inhibited surface. I am unsure why a large part of the results was dedicated to the Langmuir isotherm if all the authors were going to use it for was co show that weight loss, tafel and EIS results were all in good agreement. There were values from the electrochemical data that were tabulated but never used or explained.
A few specific comments and questions in the paper are:

· Why was no chemical cleaning done on the weight loss samples after exposure. The manuscript says only mechanical cleaning was performed. The standard practice to is chemically clean the corrosion product, as per ASTM G1.

· With the formula for "deep index", I assume the authors meant corrosion depth. The formula given is a bit confusing, as it is essentially a corrosion rate formula, as per ASTM G1. The value of the constant is given as 8,760 or 8.760. according to ASTM G1 is should be 8.76x10^4 ( = 87600) for mm/yr So either way it is wrong.

· With Table 1, why were no values given for the "blank" for Vd?

· Why have the value of inhibition coefficient (y) when the inhibition efficiency (n) was given?

· In the Results, 3.1 Wight loss method section, there is no need to state "In addition, a simple, reliableand cost-effective method does not require the use of tedious experimental processes or expensive instrumentations."

· In the same section after Table 1, the authors make the contradictory statement "With the addition of 10.0 mM of inhibitor, a rise in the mass of steel samples was observed, which unambiguously attribute this to formation of a protective film on the steel surface. Therefore, the interaction of the inhibitor molecules with the surface atoms of iron possibly lead to the formation of chemisorptions film."

· In Section 3.2.2 EIS Technique, what is meant by "… confirms higher defence…" Also that sentence needs a full stop at the end.

· Again in section 3.2.2, Table 3, how was the inhibitor efficiency calculated? I may have missed it, but it is not clear.

· In section 3.4.2 SEM Characterization, what is meant by "…shows that steel exposed to destruction in an aggressive medium…"

· There are quite a few times where there has been a space missed between words. This may not entirely be the authors fault as there may have been an issue when converting to PDF, but it needs to be addressed.

## Decision letter (RSOS-202027.R0)

This year has been very difficult for everyone, and we want to take the opportunity to thank you for your continued support in 2020.

The Royal Society Open Science editorial office will be closed from the evening of Friday 18 December 2020 until Monday 4 January 2021. We will not be responding during this time. If you have received a deadline within this time period, please contact us as soon as possible to allow us to extend the deadline. If you receive any automated messages during this time asking you to

meet a deadline, we offer apologies and invite you to respond after the festive period or during normal working hours.

With our best for a peaceful festive period and New Year, and we look forward to working with you in 2021.

Dear Dr Rakhymbay:

Manuscript ID: RSOS-202027
Title: "Synthesis and evaluation of corrosion inhibitory and adsorptive properties of N-( $\beta$ ethoxypropionitrile-N,N-bis(2-hydroxyethylethoxy) fatty amide"

Thank you for submitting the above manuscript to Royal Society Open Science. Your paper was sent to reviewers and their comments are included at the bottom of this letter.

In view of the concerns raised by the reviewers, the manuscript has been rejected in its current form. However, a new manuscript may be submitted which takes into consideration these comments.

Please note that resubmitting your manuscript does not guarantee eventual acceptance, and that your resubmission will be subject to peer review before a decision is made.

You will be unable to make your revisions on the originally submitted version of your manuscript. Instead, revise your manuscript and upload the files via your author centre.

Once you have revised your manuscript, go to https://mc.manuscriptcentral.com/rsos and login to your Author Center. Click on "Manuscripts with Decisions," and then click on "Create a Resubmission" located next to the manuscript number. Then, follow the steps for resubmitting your manuscript.

Your resubmitted manuscript should be submitted by 22-Jun-2021. If you are unable to submit by this date please contact the Editorial Office.

We look forward to receiving your resubmission.

Yours sincerely,
Dr Laura Smith
Publishing Editor, Journals

Royal Society of Chemistry
Thomas Graham House
Science Park, Milton Road
Cambridge, CB4 0WF
Royal Society Open Science - Chemistry Editorial Office

On behalf of the Subject Editor Professor Anthony Stace and the Associate Editor Professor Chaohua Cui

**********************************************

REVIEWER(S) REPORTS:
Associate Editor Comments to Author ():
RSC Associate Editor:

Comments to the Author:
(There are no comments.)

RSC Subject Editor:
Comments to the Author:
(There are no comments.)

Reviewers' Comments to Author:
Reviewer: 1

Comments to the Author(s)
This article is interested in Synthesis and evaluation of corrosion inhibitory and adsorptive properties of N-(βethoxypropionitrile-N,N-bis(2-hydroxyethylethoxy) fatty amide. There are a lot of previous studies that investigate the potential of inhibitor materials to suppress corrosion. However, the fact that the current material has not reported previously, increased the originality of this article. The techniques used in this study are classical and commonly used for the determination of the corrosion rate.
 Nowadays, theoretical studies are very important in corrosion studies but unfortunately, it is not reported in this study.
 Despite the aim of the work is to study the efficacy of the inhibitor on corrosion behavior of st3 at room temperature, but several experiments at different temperatures should be performed to determine the thermodynamic parameters.
 The introduction section is well-written but more literature should be reported in order to make a strong case for this work.
 How authors confirmed the structure of their inhibitor. Some characterization techniques should be used.
In addition, the following points should be considered:
1) A comparison between your inhibitor and some previously reported inhibitors should be included.
2) After how long the OCP equilibrium was reached?
3) The abbreviation PVD (page 6 – line 48) should be defined.
4) Space in (page 10 line 21) fora long should be corrected.

Reviewer: 2
Comments to the Author(s)
Review for RSOS-202027
Synthesis and evaluation of corrosion inhibitory and adsorptive properties of N-βethoxypropionitrile-N,N-bis(2-hydroxyethylethoxy) fatty amide

This paper reports on the corrosion testing of a vegetable oil based inhibitor on mild steel. The results presents are fairly rudimentary, and a fair bit of detail is lacking. The corrosion results mainly cover weight loss, potentiodynaic polaristaion and EIS. The surface microscopy via SEM provided no suable results other than there was corrosion on the uninhibited surface compared with the inhibited surface. I am unsure why a large part of the results was dedicated to the Langmuir isotherm if all the authors were going to use it for was co show that weight loss, tafel and EIS results were all in good agreement. There were values from the electrochemical data that were tabulated but never used or explained.
A few specific comments and questions in the paper are:
• Why was no chemical cleaning done on the weight loss samples after exposure. The manuscript says only mechanical cleaning was performed. The standard practice to is chemically clean the corrosion product, as per ASTM G1.

• With the formula for "deep index", I assume the authors meant corrosion depth. The formula given is a bit confusing, as it is essentially a corrosion rate formula, as per ASTM G1. The value of the constant is given as 8,760 or 8.760. according to ASTM G1 is should be 8.76x10^4 ( = 87600) for mm/yr So either way it is wrong.
• With Table 1, why were no values given for the "blank" for Vd?
• Why have the value of inhibition coefficient (y) when the inhibition efficiency (n) was given?
• In the Results, 3.1 Wight loss method section, there is no need to state "In addition, a simple, reliableand cost-effective method does not require the use of tedious experimental processes or expensive instrumentations."
• In the same section after Table 1, the authors make the contradictory statement "With the addition of 10.0 mM of inhibitor, a rise in the mass of steel samples was observed, which unambiguously attribute this to formation of a protective film on the steel surface. Therefore, the interaction of the inhibitor molecules with the surface atoms of iron possibly lead to the formation of chemisorptions film."
• In Section 3.2.2 EIS Technique, what is meant by "… confirms higher defence…" Also that sentence needs a full stop at the end.
• Again in section 3.2.2, Table 3, how was the inhibitor efficiency calculated? I may have missed it, but it is not clear.
• In section 3.4.2 SEM Characterization, what is meant by "…shows that steel exposed to destruction in an aggressive medium…"
• There are quite a few times where there has been a space missed between words. This may not entirely be the authors fault as there may have been an issue when converting to PDF, but it needs to be addressed.

## Author's Response to Decision Letter for (RSOS-202027.R0)

See Appendices A & B.

# RSOS-211066.R0

## Review form: Reviewer 2

**Is the manuscript scientifically sound in its present form?**
Yes

**Are the interpretations and conclusions justified by the results?**
Yes

**Is the language acceptable?**
Yes

**Do you have any ethical concerns with this paper?**
No

**Have you any concerns about statistical analyses in this paper?**
No

**Recommendation?**
Accept as is

**Comments to the Author(s)**
The authors have attempted to address all the reviewer comments.

## Review form: Reviewer 3

**Is the manuscript scientifically sound in its present form?**
Yes

**Are the interpretations and conclusions justified by the results?**
Yes

**Is the language acceptable?**
No

**Do you have any ethical concerns with this paper?**
No

**Have you any concerns about statistical analyses in this paper?**
No

**Recommendation?**
Accept with minor revision (please list in comments)

**Comments to the Author(s)**
Please check whether the "Fourier transform infra red" in the keyword is correct.

## Decision letter (RSOS-211066.R0)

We hope you are keeping well at this difficult and unusual time. We continue to value your support of the journal in these challenging circumstances. If Royal Society Open Science can assist you at all, please don't hesitate to let us know at the email address below.

Dear Dr Rakhymbay:

Title: Synthesis and evaluation of corrosion inhibitory and adsorptive properties of N-( $\beta$ ethoxypropionitrile-N,N-bis(2-hydroxyethylethoxy) fatty amide
Manuscript ID: RSOS-211066

It is a pleasure to accept your manuscript in its current form for publication in Royal Society Open Science. The chemistry content of Royal Society Open Science is published in collaboration with the Royal Society of Chemistry.

The comments of the reviewer(s) who reviewed your manuscript are included at the end of this email.

Please see the Royal Society Publishing guidance on how you may share your accepted author manuscript at https://royalsociety.org/journals/ethics-policies/media-embargo/. After publication, some additional ways to effectively promote your article can also be found here https://royalsociety.org/blog/2020/07/promoting-your-latest-paper-and-tracking-your-results/.

Thank you for your fine contribution. On behalf of the Editors of Royal Society Open Science and the Royal Society of Chemistry, I look forward to your continued contributions to the Journal.

Yours sincerely,
Dr Ellis Wilde
Publishing Editor, Journals

Royal Society of Chemistry
Thomas Graham House
Science Park, Milton Road
Cambridge, CB4 0WF
Royal Society Open Science - Chemistry Editorial Office

On behalf of the Subject Editor Professor Anthony Stace and the Associate Editor Professor Chaohua Cui.

********

RSC Associate Editor
Comments to the Author:
(There are no comments.)

*********

Reviewer(s)' Comments to Author:
Reviewer: 2

Comments to the Author(s)
The authors have attempted to address all the reviewer comments.

Reviewer: 3

Comments to the Author(s)
Please check whether the "Fourier transform infra red" in the keyword is correct.

# Appendix A

Reviewers' Comments to Author:
Reviewer: 1

Comments to the Author(s)
This article is interested in Synthesis and evaluation of corrosion inhibitory and adsorptive properties of N-(βethoxypropionitrile-N,N-bis(2-hydroxyethylethoxy) fatty amide. There are a lot of previous studies that investigate the potential of inhibitor materials to suppress corrosion. However, the fact that the current material has not reported previously, increased the originality of this article. The techniques used in this study are classical and commonly used for the determination of the corrosion rate.
¬ Nowadays, theoretical studies are very important in corrosion studies but unfortunately, it is not reported in this study.

**We agree with your valuable comment. Respectively correction was done as shown in the text of the manuscript: Nowadays, theoretical studies are very important in corrosion studies, in this work inhibitor for steel corrosion, a waste vegetable oil as a source of free fatty acid and functionalized with tris-(2-hydroxyethyl) amine, maleic anhydride and acrylonitrile as components were investigated.**

¬ Despite the aim of the work is to study the efficacy of the inhibitor on corrosion behavior of st3 at room temperature, but several experiments at different temperatures should be performed to determine the thermodynamic parameters.

**We partially agree with your comment. The investigation of temperature effect is important and gives opportunity to determine thermodynamic parameter such as activation energy of the process. The activation energy easily could be determined from the linear dependence of corrosion current from 1/T [1]. This work is focused on investigation of adsorption mechanism of selected corrosion inhibitors on the surface of the steel electrode. Knowing the magnitude of activation energy makes possible to predict corrosion rate at different (here we mean that temperature region which were not investigated for getting the dependence Jcorr vs 1/T ) temperatures and determine the limiting stage of electrode reaction. We decided study the temperature effect in further investigations**

[1] M. Boudalia, R.M. Fernández-Domene, M. Tabyaoui, A. Bellaouchou, A. Guenbour,J. García-Antón. 2019. Green approach to corrosion inhibition of stainless steel in phosphoric acid of Artemesia herba albamedium using plant extract. *Journal of Materials Research and Technology*. 8. (doi.org/10.1016/j.jmrt.2019.09.045)

¬ The introduction section is well-written but more literature should be reported in order to make a strong case for this work.

**We agree with your comment. Correction was done in the text of the manuscript**

¬ How authors confirmed the structure of their inhibitor. Some characterization techniques should be used.

**It is known from the literature that the structure of the inhibitor can be determined by NMR and FTIR analysis [2]. Unfortunately, we do not have opportunity to do NMR measurements. We tried to use FTIR.**

[2] Ahmad S, Zafar F, Sharmin E, Garg N, Kashif M. 2012 Synthesis and characterization of corrosion protective polyurethanefattyamide/silica hybrid coating material. *Prog. Org. Coatings* **73**, 112–117. (doi:10.1016/j.porgcoat.2011.09.007)

In addition, the following points should be considered:
1) A comparison between your inhibitor and some previously reported inhibitors should be included.
**Correction was done.**

2) After how long the OCP equilibrium was reached?

**OCP equilibrium was reached at exposure time equal to 120 s. Correction were done in experimental part**

3) The abbreviation PVD (page 6 – line 48) should be defined.

**Correction was done**. **Abbreviation PVD is error, it is potentiodynamic polarization PDP**

4) Space in (page 10 line 21) fora long should be corrected.

**Correction was done**

# Appendix B

Reviewer: 2

Comments to the Author(s)
Review for RSOS-202027
Synthesis and evaluation of corrosion inhibitory and adsorptive properties of N-βethoxypropionitrile-N,N-bis(2-hydroxyethylethoxy) fatty amide

This paper reports on the corrosion testing of a vegetable oil based inhibitor on mild steel. The results presents are fairly rudimentary, and a fair bit of detail is lacking. The corrosion results mainly cover weight loss, potentiodynaic polaristaion and EIS. The surface microscopy via SEM provided no suable results other than there was corrosion on the uninhibited surface compared with the inhibited surface. I am unsure why a large part of the results was dedicated to the Langmuir isotherm if all the authors were going to use it for was co show that weight loss, tafel and EIS results were all in good agreement. There were values from the electrochemical data that were tabulated but never used or explained.

**In comparative corrosion tests, methods for determining corrosion damage to metals and alloys are used: chemical and electrochemical**
**methods - in accordance with modern scientific achievements of the science of corrosion and generally accepted international (ISO) and national (NACE, DIN, ASTM, etc.) standards and GOSTs.**

A few specific comments and questions in the paper are:

• Why was no chemical cleaning done on the weight loss samples after exposure. The manuscript says only mechanical cleaning was performed. The standard practice to is chemically clean the corrosion product, as per ASTM G1.

**Yes, we agree with your comment, chemical and electrochemical polishing is used to clean the surface of the samples. The advantage of these two types of polishing is the removal of the deformed work-hardened surface layer, which increases the surface uniformity. However, these types of processing can distort the original structure of the metal due to the uneven effect of the etchant on its components.**

**Mechanical polishing - fine treatment of a pre-ground surface that produces a surface with a high degree of uniformity.**

• With the formula for "deep index", I assume the authors meant corrosion depth. The formula given is a bit confusing, as it is essentially a corrosion rate formula, as per ASTM G1. The value of the constant is given as 8,760 or 8.760.

**Correction was done**

according to ASTM G1 is should be 8.76x10^4 ( = 87600) for mm/yr So either way it is wrong.
**We don't agree with this comment. If $cm^2$ should be 10^4. Corrosion rate was calculated using unit of the surface area - $m^2$**

• With Table 1, why were no values given for the "blank" for Vd?

**Yes, we agree with your comment. Correction was done**

• Why have the value of inhibition coefficient (y) when the inhibition efficiency (n) was given? **Correction was done**

• In the Results, 3.1 Wight loss method section, there is no need to state "In addition, a simple, reliable and cost-effective method does not require the use of tedious experimental processes or expensive instrumentations." **We agree. Correction was done in manuscript.**

• In the same section after Table 1, the authors make the contradictory statement "With the addition of 10.0 mM of inhibitor, a rise in the mass of steel samples was observed, which unambiguously attribute this to formation of a protective film on the steel surface. Therefore, the interaction of the inhibitor molecules with the surface atoms of iron possibly lead to the formation of chemisorptions film."
• In Section 3.2.2 EIS Technique, what is meant by "… confirms higher defence…" Also that sentence needs a full stop at the end. **Correction was done**

• Again in section 3.2.2, Table 3, how was the inhibitor efficiency calculated? I may have missed it, but it is not clear.

**The inhibitor efficiency calculated using equation (5).**

• In section 3.4.2 SEM Characterization, what is meant by "…shows that steel exposed to destruction in an aggressive medium…"

**Correction was done**
• There are quite a few times where there has been a space missed between words. This may not entirely be the authors fault as there may have been an issue when converting to PDF, but it needs to be addressed.

**Correction was done**
